# Point of care ultrasound as initial diagnostic tool in acute dyspnea patients in the emergency department of a tertiary care center: diagnostic accuracy study

**DOI:** 10.1186/s12245-022-00430-8

**Published:** 2022-06-13

**Authors:** Himanshi Baid, Nagasubramanyam Vempalli, Subodh Kumar, Poonam Arora, Rohit Walia, Udit Chauhan, Krishna Shukla, Aakash Verma, Hannah Chawang, Disha Agarwal

**Affiliations:** 1 Department of Emergency Medicine, All India Institute of Medical Sciences Rishikesh, Rishikesh, Uttarakhand 249203 India; 2 Department of Pulmonary Medicine, All India Institute of Medical Sciences Gorakhpur, Gorakhpur, Uttar Pradesh 273008 India; 3 Department of Cardiology, All India Institute of Medical Sciences Bhatinda, Rishikesh, Punjab 151001 India; 4 Department of Radiodiagnosis, All India Institute of Medical Sciences Rishikesh, Rishikesh, Uttarakhand 249203 India; 5 Department of Emergency Medicine, Mahatma Gandhi Medical College & Hospital, Jaipur, Rajasthan 302022 India; 6 All India Institute of Medical Sciences Rishikesh, Rishikesh, Uttarakhand 249203 India

**Keywords:** Dyspnea, Emergency department, PoCUS, Point of care ultrasound, Bedside ultrasound, Diagnostic accuracy

## Abstract

**Background:**

Dyspnea is one of the common symptoms patients present to the emergency department (ED). The broad spectrum of differentials often requires laboratory and radiological testing in addition to clinical evaluation, causing unnecessary delay. Point of care ultrasound (PoCUS) has shown promising results in accurately diagnosing patients with dyspnea, thus, becoming a popular tool in ED while saving time and maintaining safety standards. Our study aimed to determine the utilization of point of care ultrasound in patients with acute dyspnea as an initial diagnostic tool in our settings.

**Methodology:**

The study was conducted at the emergency department of a tertiary healthcare center in Northern India. Adult patients presenting with acute dyspnea were prospectively enrolled. They were clinically evaluated and necessarily investigated, and a provisional diagnosis was made. Another EP, trained in PoCUS, performed the scan, blinded to the laboratory investigations (not the clinical parameters), and made a PoCUS diagnosis. Our gold standard was the final composite diagnosis made by two Emergency Medicine consultants (who had access to all investigations). Accuracy and concordance of the ultrasound diagnosis to the final composite diagnosis were calculated. The time to formulate a PoCUS diagnosis and final composite diagnosis was compared.

**Results:**

Two hundred thirty-seven patients were enrolled. The PoCUS and final composite diagnosis showed good concordance (*κ* = 0.668). PoCUS showed a high sensitivity for acute pulmonary edema, pleural effusion, pneumothorax, pneumonia, pericardial effusion, and low sensitivity for acute exacerbation of chronic obstructive pulmonary disease (AECOPD) and acute respiratory distress syndrome (ARDS)/acute lung injury (ALI). High overall specificity was seen. A high positive predictive value for all except left ventricular dysfunction, pericardial effusion, non-cardiopulmonary causes of dyspnea, and a low negative predictive value was seen for pneumonia. The median time to make a PoCUS diagnosis was 16 (5–264) min compared to the 170 (8–1346) min taken for the final composite diagnosis. Thus, time was significantly lower for PoCUS diagnosis (*p* value <0.001).

**Conclusion:**

By combining the overall accuracy of PoCUS, the concordance with the final composite diagnosis, and the statistically significant reduction in time taken to formulate the diagnosis, PoCUS shows immense promise as an initial diagnostic tool that may expedite the decision-making in ED for patients’ prompt management and disposition with reliable accuracy.

**Supplementary Information:**

The online version contains supplementary material available at 10.1186/s12245-022-00430-8.

## Background

Dyspnea is one of the familiar disturbing and debilitating symptoms patients present to the emergency department (ED) [1]. The incidence of patients coming to the ED with the chief complaint of dyspnea has been variable in various studies ranging from 0.9 to 7.4% in different regions [2–4] with an incidence of 5% in the Asia Pacific region [2].

The American Thoracic Society defines dyspnea as “a subjective experience of breathing discomfort that consists of qualitatively distinct sensations that vary in intensity” [1]. The spectrum of disorders presenting with dyspnea as the chief complaint is broad. Hence, prompt diagnosis is needed to streamline these patients' appropriate management and disposition from the ED. The subjectivity of the symptom, multiple overlapping clinical conditions for causing dyspnea, and comorbidities add to the difficulty in accurately diagnosing these patients. Initial misdiagnoses can lead to an increased hospital stay and are associated with higher mortality [5]. A focused history and physical examination often lead to the correct diagnosis; however, in 30–50% of cases, the help of more diagnostic tests may be required [6]. The dyspneic patient is often evaluated initially with a chest radiograph (CXR) and sometimes may also require chest CT (CCT) scans subsequently. These techniques expose the patient to radiation and are not feasible in pregnant patients. They have limited use in critically ill patients and depend on the resources available at the institutes (especially CCT). Thus, an early diagnostic tool in the ED to diagnose and initiate targeted management is the need of the hour.

Ultrasonography (USG) as an imaging diagnostic modality has been in clinical practice for more than 50 years [7]. However, two significant challenges limited its use in the emergency department—the earlier concept of consultative ultrasound and the limited role of the ultrasound in diagnosing respiratory disorders due to the presence of artifacts. Recently, the use of lung ultrasound in critically ill patients for diagnosis in various situations like acute respiratory failure, undifferentiated hypotension, and guiding treatment like fluid therapy has been firmly established in the form of Bedside Ultrasound in Emergency (BLUE) protocol, Rapid Ultrasonography in Shock (RUSH) protocol and Fluid Administration Limited By Lung Sonography (FALLS) protocol [8–10]. Similarly, emergency echocardiography performed by emergency physicians has also been seen to add vital information regarding patients with acute dyspnea with an overall accuracy of 97.5% [11].

There is increasing evidence supporting lung ultrasound, emergency echocardiography, and IVC assessment using ultrasound as a diagnostic tool in different specialty and clinical settings. Due to the need for prompt diagnosis in the ED, focused multifaceted ultrasound as a point of care tool is increasingly researched and incorporated by Emergency physicians. However, most of these studies have focused on identifying cardiovascular causes of acute dyspnea [12, 13]. Kajimoto et al. studied the potential of lung-cardiac-inferior vena cava (LCI) integrated ultrasound for differentiating acute heart failure syndromes (AHFS) from primary pulmonary disease in patients with acute dyspnea in the emergency setting [12, 13].

The domains of differentiating between cardiovascular and pulmonary pathologies and further differentiation of individual pulmonary pathologies remain yet to be explored.

Zanobetti et al. studied the role of PoCUS in evaluating patients presenting with acute dyspnea in the ED and tried to explore the same [14]. However, the various differentials’ distribution has also been different as per local incidence of disease; hence, uniform application of already available literature may not be applicable in every setup. Moreover, the ultrasound protocol has been different, causing different results in various studies according to the institutional protocol [14–17].

Only one study has been conducted in the South Indian subcontinent by Guttikonda et al. that determined the diagnostic performance of focused multiorgan USG in evaluating patients presenting with undifferentiated dyspnea as a chief complaint to the ED, hence leaving scope for further research for uniform application to the local population [18].

Our observational study aimed to determine the diagnostic accuracy of PoCUS in various causes of acute onset dyspnea and calculate any time benefit seen in the diagnosis and decision for disposition than the traditional methods of reaching the same in the emergency department of a tertiary care center in Northern India.

## Methods

### Study design and settings

This prospective observational study was conducted in the Emergency Department of All India Institute of Medical Sciences, Rishikesh, Uttarakhand, between November 2019 and April 2021 (patient recruitment period from January 2020 to January 2021).

### Selection of patients

#### Inclusion criteria

1. The chief complaint of acute onset shortness of breath
2. Age group: greater than 18 years of age

#### Exclusion criteria

1. Individuals referred from an outside hospital with a known diagnosis
2. Dyspnea due to traumatic cause
3. Pregnant individuals

### Sample size

All patients presenting to the ED with acute onset dyspnea were included if they met the inclusion criteria during the study’s recruitment phase. A total of 237 patients were enrolled.

### Clinical evaluation

The enrolling emergency physician recorded the patient’s medical history, vital signs, and systemic examination. The patient then was planned for relevant routine tests (chest x-ray, ECG, CBC, KFT, etc.) as deemed fit by the primary treating emergency physician. A provisional diagnosis was made from the provided list of differentials by the treating emergency physician.

### Ultrasound protocol

Each point of care ultrasound examination was performed with a multiprobe machine (SONOSITE M Turbo) by following a systematic, standardized sequence—lung ultrasound, transthoracic echocardiography, and inferior vena cava (IVC) evaluation according to a predefined ultrasound protocol.

### Lung ultrasound

It was performed with a 6- to 13-MHz linear probe. The lungs were examined by using longitudinal scans on the anterolateral aspect as per the BLUE protocol [8] and posterior thoracic area—between the posterior axillary line and spine The anterolateral examination was performed with the patient in the supine or near-to-supine position; whenever possible, dorsal areas were scanned in the sitting position or by turning the patient in the lateral decubitus on both sides in case of forced supine position.

Lung examination was targeted to detect specific ultrasound patterns identified according to international recommendations for pulmonary edema, pneumonia, pleural effusion, pulmonary embolism, acute exacerbation of chronic obstructive pulmonary disease (AECOPD)/bronchial asthma, pneumothorax, and acute respiratory distress syndrome (ARDS)/acute lung injury (ALI).

Pulmonary edema was diagnosed with the bilateral diffuse alveolar syndrome (presence of multiple B lines throughout the entire pulmonary surface symmetrically) [8].

Pneumonia was diagnosed by the presence of either pleural shredding, irregular pleural line, lung consolidation, and air bronchogram(s) with or without the focal interstitial syndrome [8].

Pleural effusion was diagnosed by an anechoic space between the parietal and visceral pleura, confirmed with the presence of a thoracic spine sign [8].

Pulmonary embolism was considered with two or more triangular or rounded pleural-based lesions indicating a pulmonary infarction or absence of any lung findings in the presence of suggestive history and evidence of RV strain [14].

AECOPD/asthma was diagnosed in the absence of any pattern mentioned above or presence of A-lines and lung sliding in a patient with suggestive medical history [8, 14].

Pneumothorax was diagnosed as the absence of lung sliding, B lines, and lung pulse with the presence of lung point [8].

ARDS/ALI was diagnosed as subpleural anterior consolidations with the absence or reduction of lung sliding, spared areas of normal parenchyma, pleural line abnormalities such as irregularly thickened or fragmented pleural line, and non-homogeneous distribution of B lines [8, 14].

### Transthoracic echocardiography

Transthoracic echo was performed with a 1- to 5-MHz curved array probe to visualize the heart in two windows—an apical four-chamber view and a subcostal long-axis view.

The assessment was done for qualitative estimation of left ventricular ejection fraction by eye-balling method, right ventricular strain, pericardial effusion, acute coronary syndrome (ACS), pulmonary embolism, any visible left ventricular hypertrophy, and valvular abnormalities.

Right ventricular strain was diagnosed with the presence of right ventricular dilation (right/left ventricular end-diastolic diameter ratio > 1 at the level of atrioventricular valve annulus).

ACS was diagnosed indirectly in the presence of regional wall motion abnormalities (RWMA) in the form of any hypokinesia or dyskinesia of the left ventricular wall in the apical four-chamber view [37].

### IVC sonography

IVC was evaluated using the 2–5-MHz curvilinear probe. The maximum and minimum diameters and the IVC collapsibility index were measured in the subcostal view in M-mode at 2 cm from the right atrial junction. The IVC collapsibility index was considered reduced if < 50%, normal, or increased if more than 50%.

The consolidated findings were recorded on a standardized form for each patient.

### Data collection

The study investigators were Emergency Medicine Residents who had received training in PoCUS for 2 months. For all eligible patients, the initial treating emergency physician was clinically evaluated in detail, and investigations were ordered as necessary. A provisional diagnosis was made, initial treatment started, and one study investigator was informed. The study investigator then performed PoCUS, blinded to the laboratory investigations but not to the clinical evaluation parameters, and made a provisional diagnosis based on them and the findings of PoCUS. The information was then given to the treating physician about the results of PoCUS. For each patient, the time of entry to the ED, the time at the end of the performance of the PoCUS, and the time to formulate the final composite diagnosis were recorded. Additional clinical parameters, final diagnosis, and patient outcome were obtained retrospectively and noted. Ultimately, the ultrasound diagnosis was compared with the final composite diagnosis (the gold standard) for accuracy and benefit in time. The final composite diagnosis was formulated by two Emergency Medicine consultants, who had access to the history, examination, and investigations performed on the patient during the ED stay. Up to four concurrent diagnoses were permitted to be made for each patient.

**Fig. 1:**
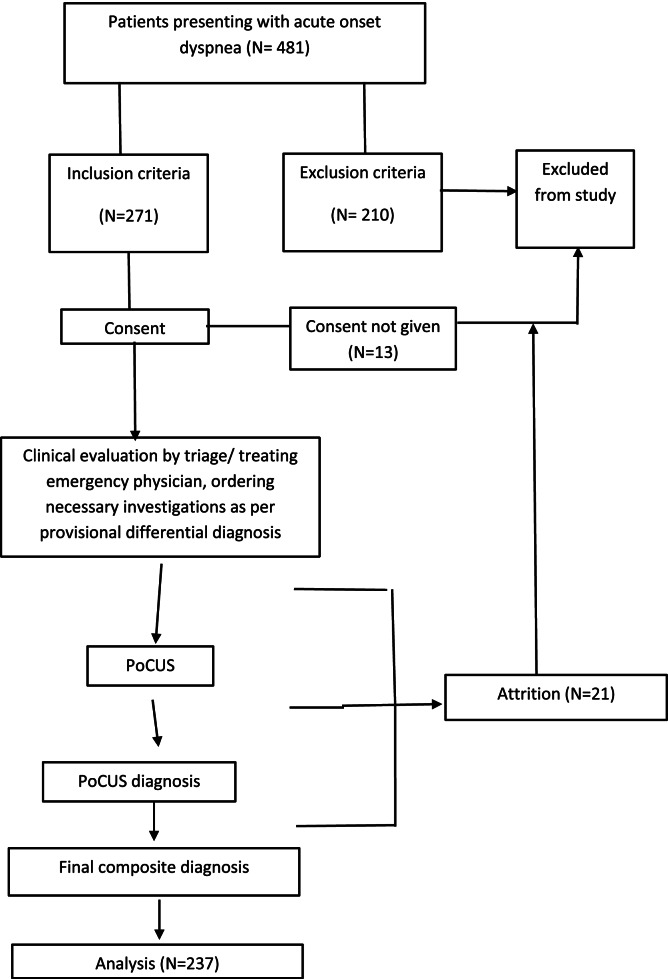
The study flow

The study flow can be depicted in Fig. 1.

### Statistical analysis

The data was entered in an Excel sheet and analyzed with the help of SPSS software Version 23.

Categorical variables were presented in number and percentage (%), and continuous variables were presented as mean (SD) and median (IQR) depending on the distribution of the data. Sensitivity and specificity analysis was done to study the diagnostic accuracy of PoCUS with the gold standard keeping a confidence interval of 95%. Kappa statistics was used to measure the concordance of the PoCUS diagnosis and the final composite diagnosis. Paired T-test was applied to compare the time taken to make the PoCUS diagnosis and final diagnosis.

## Results

Four hundred eighty-one patients with acute onset dyspnea as their chief complaint were evaluated in the ED. Two hundred forty-four were excluded from the study, and 237 patients were finally analyzed after meeting the inclusion criteria, taking informed consent, and attrition.

### Baseline characteristics of the study population

The study population’s main baseline characteristics and comorbidities are shown in Table 1.

**Table 1:** Baseline characteristics of the study population

**Characteristics**	**Values**
Age in years, median (minimum-maximum)	53 (18–82)
Men, number (%)	142 (60)
Women, number (%)	95 (40)
SBP in mm Hg, mean (standard deviation)	127.36 (32.59)
DBP in mm Hg, mean (standard deviation)	79.12 (19.46)
Heart rate in beats/min, mean (standard deviation)	105.78 (22.31)
Respiratory rate in-breath/min, mean (standard deviation)	26.21 (4.61)
Body temperature in °F, mean (standard deviation)	99.05 (0.81)
Oxygen saturation in %, mean (standard deviation)	83 (15)
Patients with sinus rhythm, number (%)	215 (91)
**Comorbidities**	**Number (%)**
None	101(43.9)
Coronary artery disease	15 (6.33)
Chronic obstructive pulmonary disease	13 (5.49)
Bronchial asthma	1 (0.42)
Diabetes mellitus	64 (27)
Hypertension	73 (30.80)
Chronic kidney disease	13 (5.49)
Malignancy	8 (3.38)
HIV	1 (0.42)
Interstitial lung disease	1(0.42)
Tuberculosis	4 (1.69)
Chronic liver disease	1 (0.42)
Hypothyroidism	7 (2.95)

### Ultrasound findings

Of the 237 PoCUS done, 27 (11.4%) of the scans were normal, while 210 (88.6%) were abnormal for pathology. The different findings of ultrasound findings are represented in Table 2.

**Table 2:** Lung and cardiac ultrasound findings (*n*=237)

	Present number (%)
**Lung ultrasound variables**	
Absence of lung sliding	2 (0.84)
Unilateral pleural effusion	14 (5.91)
Bilateral pleural effusion	19 (8.12)
Diffuse B lines (by counting)	71 (29.96)
Grouped B lines (by counting)	70 (29.54)
Irregular pleura	157 (66.24)
Pleural shredding	132 (55.70)
**Cardiac ultrasound variables**	
Decreased ejection fraction	14 (5.91)
Right ventricular strain	7 (2.95)
Pericardial effusion	3 (1.28)
Regional wall motion abnormalities	1 (0.42)
Others	25 (10.55)
All chamber dilatation	2
Global hypokinesia	4
Left ventricular hypertrophy	13
Valvular abnormality	5
Dextrocardia	1

### Causes of dyspnea and diagnostic accuracy of PoCUS

The causes of acute onset dyspnea according to ultrasound diagnosis and final composite diagnosis are reported in Table 3. In our study group, the most common final composite diagnosis was pneumonia (*n*=188), followed by acute pulmonary edema (*n*=35) and ARDS/ALI (*n*=35 each). The diagnostic performance of ultrasound was compared for each specific disease with the final composite diagnosis, shown in Additional file 1.

**Table 3:** Causes of dyspnea according to ultrasound diagnosis and final composite diagnosis

Variable	No. of patients with ultrasound diagnosis	No. of patients with final composite diagnosis
Pneumonia	167	188
Acute pulmonary edema	37	35
Pleural effusion	21	16
LV dysfunction	14	9
ARDS/ALI	11	35
Pericardial effusion	3	2
AECOPD/asthma	1	16
Acute coronary syndrome	1	2
Pulmonary embolism	1	1
Pneumothorax	1	1
**Others**	**41**	**29**
Normal/inconclusive	27	0
Valvular disease	5	4
DCM	2	2
RV strain	7	0
Acute anxiety attack	0	1
Airway obstruction	0	3
Malignancy	0	10
Angina equivalent	0	1
Myasthenia crisis	0	1
DKA	0	1
Metabolic acidosis	0	1
Complete heart block	0	1
Upper respiratory tract infection	0	3
Severe PAH	0	1
Total	298	334

### Concordance between PoCUS and final composite diagnosis

The concordance was categorized as optimal with a kappa value between 0.8 and 1, good with a kappa value between 0.6 and 0.79, moderate with a kappa value between 0.4 and 0.59, and poor kappa values less than 0.4. The overall concordance was good (*κ* =0.668) between the ultrasound and final composite diagnosis. The detailed concordance is depicted in Table 4.

**Table 4:** Concordance between PoCUS diagnosis and final composite diagnosis

Diagnosis	***K*** value
Pneumonia	0.634
Acute pulmonary edema	0.836
Pleural effusion	0.854
LV dysfunction	0.590
Pericardial effusion	0.798
ACS	0.665
Pulmonary embolism	1.000
Pneumothorax	1.000
AECOPD/asthma	0.111
ARDS/ALI	0.392
Others	0.466
**Total**	**0.668**

### Comparison of time taken to formulate diagnosis using PoCUS and traditional methods in the ED

The median time for PoCUS diagnosis was 16 min, with a minimum of 5 min and a maximum of 264 min. The median time for making the final composite diagnosis was 170 min, with a minimum of 8 min and a maximum of 1346 min. This showed that the time taken for PoCUS diagnosis was much lesser when compared to the traditional methods of formulating the ED diagnosis for disposition. On comparing the two, using the paired *T*-test, the results were statistically significant with a *P* value of <0.001.

## Discussion

Our study population comprised 237 patients with a median age of 53 years (18–82 years), with 40% female patients. Diagnostic accuracy studies were done in European countries and the USA, in dyspnea patients had shown a population distribution with a higher mean age group and comparable gender distribution [14, 15]. The only similar study published in the Indian setting by Guttikonda et al. enrolled 108 patients of undifferentiated dyspnea with a demographic distribution of age similar to our study. The mean age was 50+/−15.85 (16–90 years). The gender distribution was, however, not reported [18].

The most common final composite diagnosis in our study population was pneumonia (*n*=188, 79.32%). However, the most common diagnosis and proportional distribution of differentials for acute dyspnea differed in different regions [14, 18]. The sensitivity and specificity of PoCUS were per previously reported literature. Our study showed very similar sensitivity and specificity (sensitivity 85.6% vs. 88% and specificity 87.7% vs. 88%) in comparison to a meta-analysis done in 2017 by Ling Long et al. The positive likelihood ratio (6.99 vs.5.37) and negative likelihood ratio (0.16 vs. 0.13) were also comparable to the pooled data [19]. It was also similar to the study conducted later by Zanobetti et al. (sensitivity 85.6% vs. 88.5% and specificity 87.7% vs. 91.6%), where the gold standard was the final diagnosis made by Emergency Medicine experts who had access to all patient information during their hospital stay [14]. However, PoCUS cannot rule out pneumonia diagnosis due to its low negative predictive value (61.4%) as per our findings. The subsequent commonest diagnosis was acute pulmonary edema (*n*=35, 14.76%). The sensitivity was lower in our study when compared to the existing literature (88.5% vs. 97%), while the specificity was similar to the literature (97.7% vs. 98%) [20]. The low sensitivity seen could be attributed to less specific diagnostic criteria for acute pulmonary edema (in the form of a number of B lines, distribution of the same in a specific number of windows) in our study and different diagnostic criteria in previous studies. Also, our study investigators were Emergency Medicine Residents trained for only 2 months, thereby leaving a learning curve to reach expertise. The addition of the transthoracic echocardiography as a component of PoCUS in our study to detect LV dysfunction (sensitivity of 77.7% and specificity of 96.9%) or ACS (sensitivity 50% and specificity 100%) further will help in narrowing down the cause of acute pulmonary edema and streamline management of the patient. The high negative predictive value of 98% shows that PoCUS can be reliably used as an initial tool for ruling out acute pulmonary edema diagnosis.

For diagnosing pleural effusion, the sensitivity and specificity were higher in our study group than in a recent meta-analysis by Hansell et al. (sensitivity 100% vs.91% and specificity 97.7% vs. 92%), where CT scan was considered the gold standard [21]. The difference between the two owed to different gold standards. Not all patients underwent CT scans in the ED to formulate a diagnosis for ED disposition in our study. The relatively lower positive predictive value of 76.1% cannot make PoCUS a reliable initial tool for ruling in pleural effusion. Still, according to our study, a high negative predictive value of 100% makes it a reliable tool for ruling out pleural effusion diagnosis.

For the diagnosis of ARDS/ALI, the sensitivity of lung ultrasound has been variable depending on the difference in the lung ultrasound protocols used, the regions evaluated for the set findings, the defining criteria for ARDS, and the gold standard used for comparison [14, 22, 23]. In our study, the sensitivity (28.5%) was very low, while the specificity was high (99.5%). This was due to overlapping ultrasound features in pneumonia and ARDS/ALI, making it difficult to make the concurrent diagnosis of both in the PoCUS performed. Our study derived that the positive predictive value of 90.9 and negative predictive value of 88.9 makes PoCUS a reliable diagnostic tool for ruling in the diagnosis of ARDS/ALI. However, it may not be easily detected in the initial phases, where it may be confused for pneumonia and when associated with pneumonia.

For the diagnosis of LV dysfunction, our study showed a sensitivity of 77.7% and a specificity of 96.9%. According to previous studies, focused echocardiography performed by trained emergency physicians has shown high agreement rates with cardiologists (detection of LV dysfunction was seen to be 82.6%) [24]. But, results from our study cannot be compared to the same due to the difference in the measurement variables. However, the eye-balling method for evaluating LV dysfunction was used in our protocol which is a subjective assessment method; it may vary from observer to observer. Introducing a semi-quantitative or qualitative measure for assessing LVEF may improve the diagnosis accuracy but need a steeper learning curve and greater time.

Our study detected acute coronary syndrome by indirectly detecting regional wall motion abnormalities. An earlier study by Farsi et al. showed 100% agreement between the Emergency Physician and Cardiologist in detecting regional wall motion abnormalities [25]. In our study population, the final composite diagnosis for acute coronary syndrome was only 2, of which 1 was detected on PoCUS. The sensitivity was thus 50% and specificity 100%. NST ACS may not manifest with regional wall motion abnormality; hence, it may not be detected by PoCUS. However, due to these patients’ insufficient numbers in the study population, it is impossible to interpret our results accurately. More research is required on this topic to study the same applications in everyday practice.

Sixteen patients in our study group had a diagnosis of AECOPD/Asthma, but only 1 was detected based on PoCUS and suggestive history, reducing the sensitivity to 6.2% in our study. However, previous studies have reported a sensitivity of 89% [8] and 86.8% [14]. This gross difference was seen because the presence of concomitant pneumonia or any other abnormality in PoCUS would be interpreted as the specific abnormality. According to our diagnostic criteria, AECOPD/asthma, on ultrasound, was a diagnosis of exclusion, thereby making it difficult to diagnose by PoCUS in situations where concomitant diseases that can cause abnormalities in the scan were also present.

Although our study also showed high sensitivity and specificity for diagnosing pericardial effusion, pneumothorax, and pulmonary embolism, unfortunately, the total number of cases in our study group with these final composite diagnoses were too few making this result not applicable to the general population in these clinical conditions.

The various other diagnoses made by PoCUS for acute onset dyspnea included valvular disorders, RV strain, LV hypertrophy, and dilated cardiomyopathy.

The final composite diagnoses also included disorders like the neuromuscular cause of dyspnea (bulbar myasthenia crisis), acute airway obstruction, and psychological causes that revealed a normal PoCUS scan. The overall sensitivity and specificity of these other causes were 65.6% and 89.4%. PoCUS cannot be used for diagnosing airway obstruction, neuromuscular, or psychiatric causes of acute dyspnea; however, the role in identifying valvular disorders and other cardiac abnormalities is still explorable with improvement in the learning curve.

The overall concordance of the PoCUS diagnosis with the final composite diagnosis was good, with a kappa value of 0.668, making PoCUS a promising diagnostic tool for decision-making in the ED for acute dyspnea patients. This was comparable to the study by Zanobetti et al., where the kappa value was 0.711 for concordance of ultrasound diagnosis with ED diagnosis [14]. However, Guttikonda et al. [18] found the concordance between ultrasound diagnosis and final hospital diagnosis to be higher (*κ*=0.805), which was higher than that seen in our study. We found that the median time for formulating a PoCUS diagnosis was 16 min, with a minimum time of 5 min and a maximum of 264 min. The extensive range was due to various factors, including the time taken for informing the study investigators, the time taken by the study investigator to approach the patient, any life-saving procedure which was given preference to the workup of the patient for further management, and the availability of the portable ultrasound machine. Our ED is equipped with only one portable ultrasound machine, and patients were triaged in different areas depending upon their clinical status. The median time for formulation of the ED diagnosis was 170 min with a minimum time of 8 min and a maximum time of 1346 min, which depended on the diagnosis, the traditional methods required for making the diagnosis, the time required for shifting for the radiological investigations and running laboratory investigations where required. The time to formulate the PoCUS diagnosis was much less than required to make the final composite diagnosis. The difference was statistically significant with a *p* value of <0.001. Earlier studies have also shown a statistically significant reduction in decision-making using PoCUS in the ED for management and disposition; however, the time to make the diagnosis via traditional methods varied depending on the difference in resource availability and logistic issues unique to individual ED settings [14, 26].

## Limitations

The small sample size and the uneven distribution of differentials of acute onset dyspnea prompt similar multicentric studies catering to larger study groups. PoCUS, despite the presence of diagnostic criteria, is a subjective mode of diagnosis which may vary from observer to observer. Measurement of inter-observer variability would validate the use of PoCUS better. The gold standard in our study was final composite diagnosis was made by Emergency Medicine consultants. As they were also involved in the active management of the patent, a clinical bias could not be ruled out. Although the benefit in time comparison showed statistically significant results in our study, it could vary in different settings depending on available resources and logistics.

## Conclusion

The present study used PoCUS as an initial diagnostic tool for evaluating acute dyspnea patients in the emergency to improve and hasten accurate decision making. By combining the overall accuracy of PoCUS, the concordance with the final composite diagnosis, and the statistically significant reduction in time taken to formulate the diagnosis, PoCUS shows immense promise as an initial diagnostic tool in evaluating patients with acute dyspnea in the ED and also for facilitating quicker decision-making.

## Supplementary Information

**Additional file 1.** Diagnostic accuracy of PoCUS in comparison to final composite diagnosis.

## Data Availability

The datasets used and/or analyzed during the current study are available from the corresponding author on reasonable request.
