# Supplementary figures and images for: Point of care ultrasound as initial diagnostic tool in acute dyspnea patients in the emergency department of a tertiary care center: diagnostic accuracy study
Source: Int J Emerg Med. 2022 Jun 13;15:27. doi: 10.1186/s12245-022-00430-8 (PMC9190130; doi:10.1186/s12245-022-00430-8)

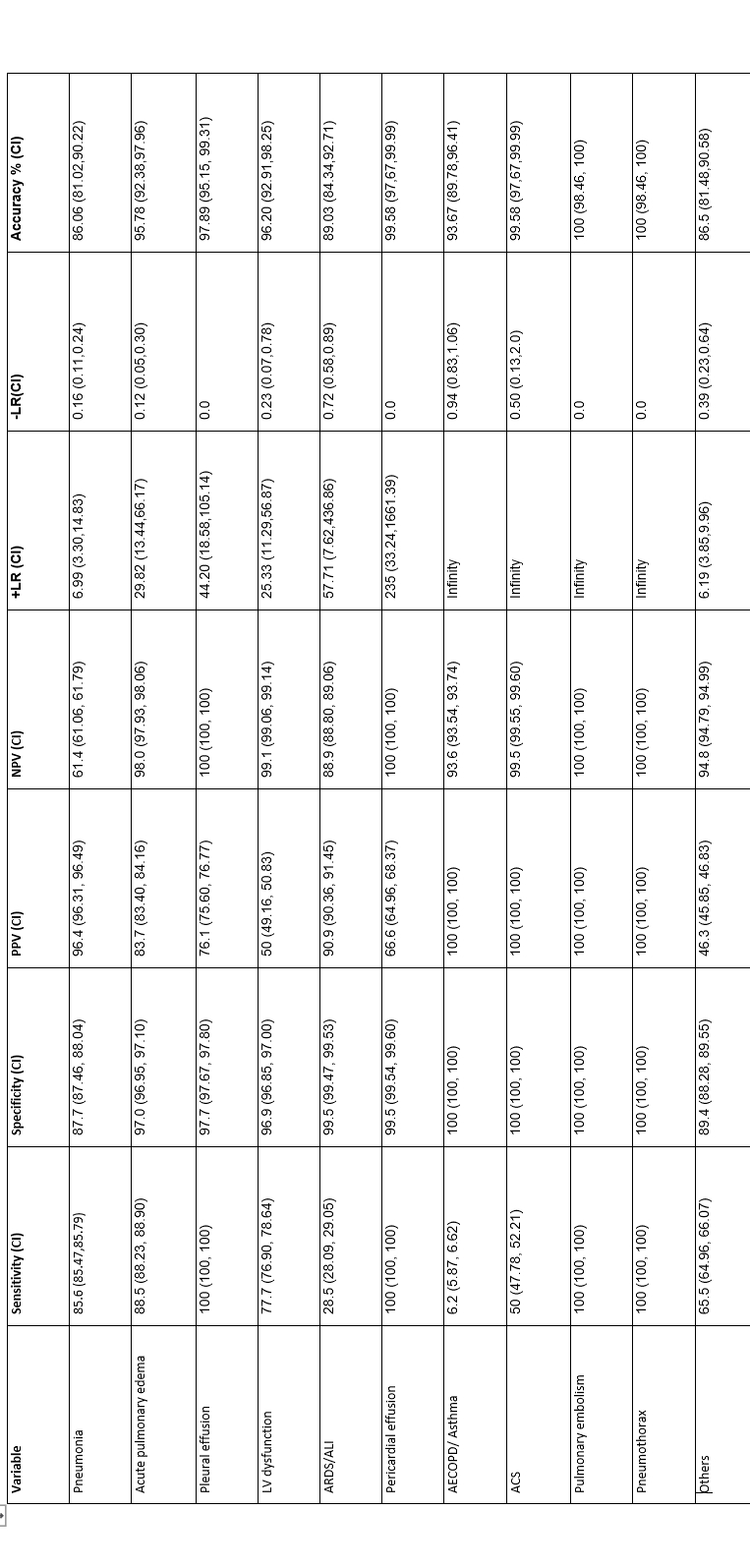

Supplement: Supplementary file 1 — Additional file 1. Diagnostic accuracy of PoCUS in comparison to final composite diagnosis. [file 12245_2022_430_MOESM1_ESM.jpg]
